# Hysteroscopic treatment of symptomatic adenomyoma

**DOI:** 10.4274/jtgga.galenos.2019.2019.0062

**Published:** 2020-06-08

**Authors:** Jin Yu, Duo Zhang, Wei Xia, Jian Zhang

**Affiliations:** 1 Department of Obstetrics and Gynecology, International Peace Maternity and Child Health Hospital School of Medicine, Shanghai Jiaotong University, Shanghai, China; 2 Institute of Embryo-Fetal Original Adult Disease Affiliated to School of Medicine, Shanghai Jiaotong University, Shanghai, China; 3 Shanghai Key Laboratory of Embryo Original Diseases, Shanghai, China

**Keywords:** adenomyoma, adenomyosis, hysteroscopy, hysteroscopic surgical procedures

## Abstract

Hysterectomy has been the definitive treatment option for symptomatic adenomyosis and/or adenomyoma when medical or other conservative treatments fail to control the symptoms. Conservative surgery has already developed as an alternative treatment because of patients’ increasing desire to preserve their uterus. This video demonstrates a novel hysteroscopic treatment of symptomatic adenomyoma for patients with no desire for fertility.

## Introduction

Adenomyosis is a kind of benign gynecologic disorder with the invasion of endometrial glands and stroma in the uterine myometrium, which results in pelvic pain, dysmenorrhea, and menorrhagia (1). The disease may be diffuse or focal with adenomyoma. Hysterectomy has been known as the primary treatment for adenomyosis and/or adenomyoma (2).

Traditionally, adenomyosis would be found incidentally in specimens obtained from uterine biopsies or hysterectomy and/or percutaneous ultrasound-based biopsies. Modern diagnostic imaging techniques, such as magnetic resonance imaging (MRI), which have high accuracy in identifying this kind of pathology, have led to conservative uterine-sparing treatments of adenomyosis and/or adenomyoma becoming efficacious and feasible (3,4).

**Figure 1:**
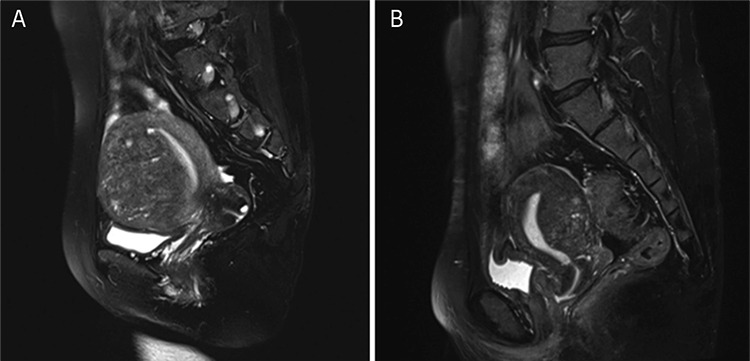
Magnetic resonance imaging imaging of adenomyosis The adenomyotic lesions in case one were located in the anterior uterine wall (A), and the lesions in case two were located in the posterior wall (B)

**Figure 2:**
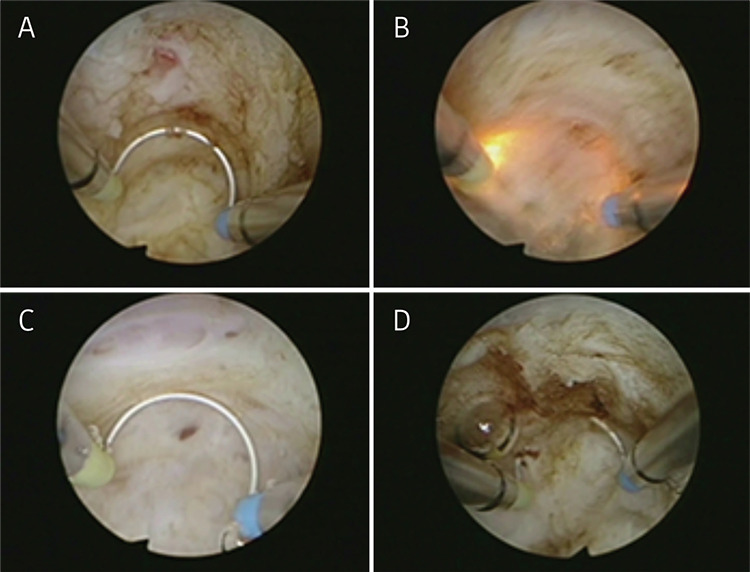
Surgical procedure In case one, upon cutting the endometrium covering the adenomyotic lesions (A), pink ectopic endometrial lesions in the myometrium were exposed. (B) The ectopic endometrium and adenomyotic lesions were gradually excised from the myometrium. (C) During the resection of lesions, several intramural microcysts with a wide base were revealed. (D) Opening the microcyst resulted in the outflow composed mostly of old blood

**Figure 3:**
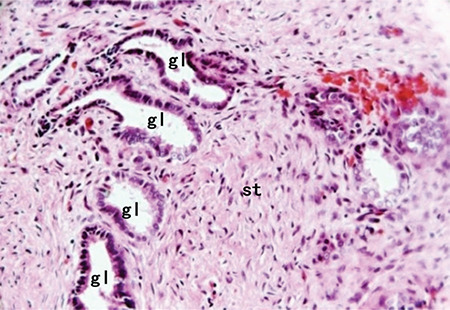
HE staining of adenomyosis St: Stromal cells, gl: Glands

This video shows the hysteroscopic surgical procedures of two women with adenomyoma (Figure 1) requesting surgical management for the relief of symptoms and the preservation of the uterus, but with no desire for future fertility. These two patients both had heavy menstrual bleeding and severe dysmenorrhea. We used saline solution to dilate the uterine cavity and set the intrauterine pressure at 120 mmHg. The operation was performed with a transcervical resection resectoscope equipped with a 3 mm and 5 mm wide loop. The surgeon dilated the cervix to 9 mm, then used a cutting loop to resect the lesions repeatedly and progressively. With color Doppler ultrasound guidance, the first step was to evaluate the features of the uterine cavity. Then, the surgeon used a cutting loop to progressively resect the lesions (Figure 2). The operation was completed with the appearance of the pink fasciculate structure of the myometrium. Tissue fragments were removed at intervals using ovum forceps. The specimens were sent for histologic analysis (Figure 3).

Follow-up was performed twice at 3-month intervals. The patient menstruated regularly. The postoperative visual analogue scale scores of menstrual blood volume and dysmenorrhea appeared to decline substantially. The uterine volume was evaluated using MRI 6 months later and was reduced by approximately 33%.

Uterine perforation is the greatest risk associated with hysteroscopic resection surgery. For the duration of the procedure, the surgeon should pay particular attention to fluid management and prepare with solutions when fluid overload or hyponatremia is suspected. Hysteroscopic excision of uterine adenomyoma has the following benefits: The uterus is preserved and the symptoms of adenomyoma are improved; the minimally invasive operation takes a short time and patients recover quickly. Therefore, hysteroscopic excision can become an effective conservative treatment option for adenomyoma.
